# The Construct and Measurement of Perceived Risk of Nonremunerated Blood Donation: Evidence from the Chinese Public

**DOI:** 10.1155/2015/302043

**Published:** 2015-10-07

**Authors:** Liangyong Chen, Zujun Ma

**Affiliations:** School of Economics and Management, Southwest Jiaotong University, Chengdu 610031, China

## Abstract

The perceived risk of nonremunerated blood donation (NRBD) is one of the most important factors which hinder the Chinese public from donating blood. To understand deeply and measure scientifically the public's perceived risk of NRBD, in this paper the qualitative and quantitative methods were used to explore the construct of perceived risk of NRBD in Chinese context. Firstly, the preliminary construct of perceived risk of NRBD was developed based on the grounded theory. Then, a measurement scale of perceived risk of NRBD was designed. Finally, the exploratory factor analysis (EFA) and confirmatory factor analysis (CFA) were adopted for testing and verifying the construct. The results show that the construct of perceived risk of NRBD has three core dimensions, namely, trust risk, psychological risk, and health risk, which provides a clear construct and concise scale to better capture the Chinese public's perceived risk of NRBD. Blood collection agencies can strategically make polices about perceived risk reduction to maximize the public's NRBD behavior.

## 1. Introduction

It is well known that blood plays an irreplaceable role in medical treatment and relies mainly on nonremunerated blood donation (NRBD). However, blood shortage has been one of the most important issues which need to be solved urgently in China, because the rate of blood donation is very low in China. Based on the statistics from the Ministry of Health (MOH) of China, the donation rate was only 9‰ of Chinese population in 2011, which is even lower than the lowest standard recommended by World Health Organization (WHO). Meanwhile, with the fast development of medical care and medical service, the blood demand in clinical medicine increases quickly. Therefore, the blood supply has become a severe challenge.

Differing from general helping behavior, NRBD behavior has its own particularity because the Chinese public believe that blood is the soul of being and the foundation of life. Despite modern medical science has proven that a moderate amount of blood donation can accelerate metabolism, the majority of the Chinese public are unwilling to donate blood. Moreover, in recent years several scandals related to public welfare result in a sharp decline of the social credibility of Chinese nonprofit organizations. For example, Guo Meimei flaunted her wealth and was claimed on Sina Weibo (known as China's twitter) to be the general manager of a company called Red Cross Commerce in 2011, which plunged the Red Cross Society of China into an unprecedented crisis of trust. The NRBD activities are also involved and oppugned by the Chinese public.

Only the public's active participation and long-term support can ensure a healthy and sustainable development of NRBD project. However, a deep-rooted prejudice against blood donation, a lack of scientific understanding of blood donation, and the distrust of the blood collection organization all give the Chinese public a false impression that NRBD is a risky activity. As Slovic [1] pointed out, little probability of the objective risk cannot stop the majority from assessing risk based on their own subjective judgments, which is called as the perceived risk. Perceived risk, being different from a real risk, is rooted in people's subjective cognition and has an important effect on human choice [2–4]. Perceived risk has been one of the most important topics in psychology and was originally defined by Bauer [5] to have two-dimensional structure, namely, uncertainty and adverse consequences.

There have been many studies that examined the salient risk facets of NRBD, such as ineffective incentive [6–8], the dislike of needles and the pain associated with them [9, 10], physical injury [6, 11], being harmful to health [12], contagion [13, 14], a fear of fainting [11, 15], disliking the sight of blood [16, 17], a concern that illness will be revealed in the screening tests associated with the blood donation [13, 18], lifestyle barriers [8, 16], and resulting in anemia [12, 13]. Some scholars also explicitly incorporated perceived risk into the studies on blood donation decision. Nonis et al. investigated whether American college students could be subdivided into blood donors and nondonors from different perspectives, namely, perceived risk, demographic characteristics, and nonmonetary incentive [19]. The results reflected that there was no difference between blood donors and nondonors in terms of perceived risk. Allen and Butler, by means of structural equation model (SEM), evaluated how the knowledge about blood donation and perceived risk affects the intention of blood donation in America [20]. Barkworth et al. used the multiattribute model to study the relationship between perceived risk and blood donation rate in England and found that perceived risk could directly affect blood donation rate [21]. These studies, however, show little consensus regarding a common set of risk facets applicable across blood donation situations.

To the best of our knowledge, the construct of perceived risk of NRBD has not been explored systematically and there is still a lack of scientific scales for measuring the perceived risk of NRBD. Moreover, the public's perceived risk of NRBD may change with the changes in the social and cultural context as time goes on. So the concept of perceived risk of NRBD is somewhat of a fuzziness. Therefore, researches on risk perception and its effect on blood donation decision were not pushed forward to the further level. What is more, perceived risk varies in different context and cultures, and foreign research conclusions cannot be completely applied to the Chinese context. However, presently there is no study on the perceived risk of NRBD conducted in Chinese context. So it is important to conceptualize and operationalize the construct of perceived risk of NRBD in Chinese social and cultural context.

**Figure 1:**
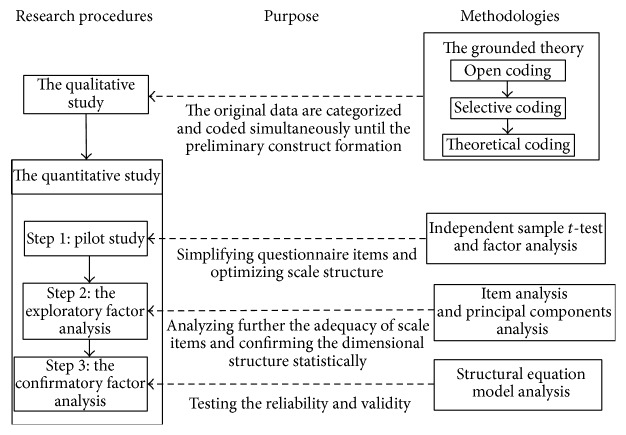
The research framework.

The purpose of this paper is hence to develop the construct of perceived risk of NRBD and conduct a scientific measurement of the construct. Firstly, the preliminary construct of perceived risk of NRBD is established based on the classical grounded theory. Then, a measurement scale for perceived risk of NRBD is designed for testing preliminarily the construct of perceived risk of NRBD based on the exploratory factor analysis (EFA). Finally, the adequacy of the construct of perceived risk of NRBD and the measurement scale are further examined by using the confirmatory factor analysis (CFA). The structural framework of this paper has been shown in Figure 1.

The results of this study may provide a clear construct to better capture the Chinese public's perceived risk of NRBD. In particular, the proposed measurement scale may provide a concise and scientific way to assess the perceived risk of NRBD, which is the precondition for exploring the effect of perceived risk on blood donation behavior. In addition, the results may be very helpful in creating effective marketing strategies for reducing the perceived risk of NRBD.

## 2. The Qualitative Study

**Figure 2:**
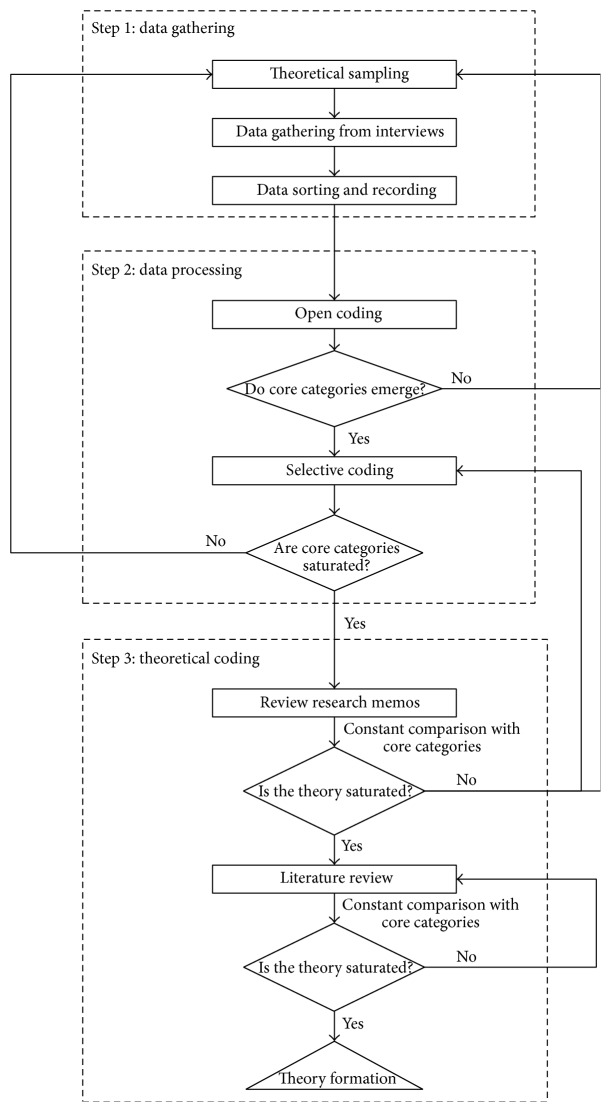
The procedure of the grounded theory.

In this paper, the classical grounded theory was employed to develop the construct of perceived risk of NRBD. The grounded theory was proposed by Glaser [22] and has been regarded as one of the most fundamental frontiers of qualitative research. The grounded theory can be used to uncover information immersed in both context and cultures so as to develop the corresponding construct. Based on the method, the original data should be gathered by means of interview and then coded openly and selectively. In this way, the construct of perceived risk of NRBD could be developed. The research procedure of the grounded theory was provided in Figure 2.

### 2.1. Sample Description

In line with the grounded theory, we gathered the data through interviews, and the data were analyzed and coded simultaneously until the theoretical saturation. Taking this as the research principle, we interviewed 83 objects, including students, soldiers, civil servants, office workers, and self-employers. The sample information of interviewees was as follows:

- Gender: male (55.4%), female (44.6%); age, 18 to 30 (27.7%), 31 to 40 (30.1%), 41 to 50 (26.5%), and 51 to 60 (15.7%).
- Educational background: junior high school level or lower (13.3%), high school level (32.5%), bachelor's degree (38.6%), and master's degree or above (15.7%).
- Income: below Ұ2000 (14.5%), Ұ2001 to Ұ4000 (40.1%), Ұ4001 to Ұ6000 (25.3%), and above Ұ6000 (19.3%).
- Profession: students (24.1%), soldiers (10.8%), farmers (12%), civil servants (7.2%), office workers (28.9%), self-employers (6%), and others (10.8%).
- Blood donation experience: yes (16.9%), no (83.1%).

### 2.2. Data Gathering

The first-hand data were gathered by means of the semistructured interview. At the same time, network search is a great supplement for the data about blood donation, which covers second-hand materials from news reports, microblogs, and forums.

The interview outline about the public's perceived risk of NRBD was designed based on the characteristics of research objects and contents. The face-to-face interview and online interview were two main methods. The interviewees were encouraged to answer the presupposed question “what risks do you think exist about nonremunerated blood donation?”. The interview processes were recorded in sound form and written form under the permission of the interviewees. Before the formal interviews, we selected ten samples to conduct pilot interviews to find out the adequacy of the interview outline and procedure and accordingly modified them based on the feedback information about the pilot interview. Then, the formal interview was conducted.

When sorting out the data, we converted recording materials into written materials and made a comparison with the original written materials. After interviewees ensured the truth of the complete written interview materials, the final materials were analyzed and coded.

### 2.3. Coding Process

#### 2.3.1. Open Coding

Open coding refers to a coding method that the gathered materials are coded line by line and the corresponding concepts are extracted, and then the data and extracted concepts are dismissed and recombined through constant comparison. In order to naturally obtain initial concepts, researchers should keep an open attitude toward study and abandon the preconceived notions in this process [25]. To avoid the intervention of researchers' opinions and inference, we used the native code as far as possible. In other words, interviewees' exact words were labeled and regarded as the initial concepts. In this study, 258 initial concepts were extracted from 71 samples.  Table 1 presents the example of open coding.

#### 2.3.2. Selective Coding

Once open coding was completed, the extracted initial concepts would be coded selectively. The ultimate goal of selective coding is to extract the core category from initial concepts [26]. The core category naturally emerges during the process of open coding and it owns the following main features: (a) the correlation, which means that the core category is associated with other data or properties, (b) high explanatory power, (c) frequent appearance, and (d) having meaningful association with other variables.

By comparing the initial concepts from open coding, we extracted the core concepts which were associated with the perceived risk of NRBD. Through comparison and screening, 258 initial concepts, in accordance with correlation and frequency, were merged into 39 subcategories, which were further merged into 3 core categories, that is, trust risk, psychological risk, and health risk, as shown in Table 2. Trust risk shows that the Chinese public has begun to oppugn the public nature of NRBD, the professionalism of blood collection staffs, and the credibility of blood collection organizations, such as the Chinese Red Cross. Psychological risk is closely related to the public's psychological state and shows the public's worry before or after blood donation. Health risk represents the blood donors' worry about whether blood donation is harmful to their health. Because of the uniqueness of source and the importance of blood rooted in Chinese culture, the effect of NRBD on health is always the public's focus.

#### 2.3.3. Theoretical Coding

Theoretical coding is aimed to further confirm the structure of the extracted core categories which present naturally in selective coding [26]. According to the procedure of the grounded theory, reviewing research memos in which the viewpoint, concept, and categories developed in the process of research are recoded and literature comparison is the key of theoretical coding [26]. First, we compared constantly the core categories extracted from the selective coding with research memos. If the theory is not saturated, we need to conduct theoretical sampling and selective coding once again to replenish new data to realize the theoretical saturation. Then, literature review was conducted. The core categories were compared constantly with available literature, as shown in Table 3, to find out the adequacies of the present theory and make further replenishment. When new concepts and categories could be no longer proposed by constant literature comparison, the theoretical coding was completed.

Based on the above research procedure, we preliminarily established the multidimensional construct of perceived risk of NRBD. The three core categories, that is, trust risk, psychological risk, and health risk, jointly construct the public's mental pattern of perceived risk of NRBD. Meanwhile, according to Table 3, it is not hard to find that the construct of perceived risk of NRBD has special Chinese characteristics, which are closely related to Chinese social and cultural context.

## 3. The Quantitative Study

Compared with other qualitative methods, the classical grounded theory can develop the construct under the specific context and culture, so it is featured with high objectivity. However, it is unavoidable for researchers to mix their own subjective cognition into the research. Given this, the multidimensional construct of perceived risk of NRBD based on the grounded theory should be further verified through exploratory factor analysis (EFA) and confirmatory factor analysis (CFA) to prove its reasonableness.

### 3.1. Pilot Study

The pilot study aims at simplifying questionnaire items and optimizing scale structure before exploratory factor analysis and confirmatory factor analysis are conducted. Based on the multidimensional construct of perceived risk of NRBD developed based on the grounded theory, we designed 39 items corresponding to the above three dimensions as the initial questionnaire items. First, item analysis was conducted as the basis for questionnaire item screening and modification. Participants' scales were arranged from the highest total score to the lowest, and the top 27% and the last 27% were selected as the extreme groups which were analyzed by the independent-sample *t*-test to identify item difference. Then, the factor analysis was conducted to calculate the factor loading value and to find out whether there exists cross-loading among items. The items with factor loading below 0.4 and cross-loading would be deleted.

#### 3.1.1. Data Gathering

The survey was conducted via a five-point Likert scale, and the items were arranged at random. 240 questionnaires were totally handed out and 224 were returned on the spot. That is, the valid response rate was 93.3%. The sample information of the pilot study was as follows:

- Gender: male (52%), female (48%); age, 18 to 30 (38.4%), 31 to 40 (29.9%), 41 to 50 (20.5%), and 51 to 60 (11.2%).
- Educational background: junior high school level or lower (10.3%), high school level (34.4%), bachelor's degree (50.9%), and master's degree or above (13.4%).
- Income: below Ұ2000 (13.8%), Ұ2001 to Ұ4000 (34.8%), Ұ4001 to Ұ6000 (38.4%), and above Ұ6000 (12.9%).
- Profession: students (16.5%), soldiers (8.9%), teachers (4%), civil servants (18.3%), office workers (45.1%), and self-employers (7.1%).
- Blood donation experience: yes (15.2%), no (84.8%).

#### 3.1.2. Data Analysis and Results

The results of item analysis showed that the score gap of all items in the high score group and the low score group reached the 0.05 significant level, while 10 items did not reach the 0.001 significant level. Further analysis indicated that 4 of 10 items can be found to have similar expressions in other items so they were combined with others, while the remaining 6 items were not expressed clearly, so they were adjusted in the formal questionnaire. The correlation coefficient of 4 items between the item score and the total score was less than 0.3. When one of items was deleted, the coefficient of internal consistency (Cronbach's alpha) increased.

The results of factor analysis showed that Kaiser-Meyer-Olkin (KMO) value, that is, the measurement of sampling adequacy of pilot samples, was 0.755 and chi-square value of Bartlett's test was 237.001 (*p* < 0.001), which indicated that the sample data were suitable for factor analysis. We explored a clear factor structure through the method of principle component analysis with the varimax rotation.

On the basis of the above analysis, the semantic duplicated items were merged, the vaguely expressed items were adjusted, and the unreasonable items were deleted. Ultimately, 34 items of 39 were included in the formal scale. It should be specially explained that some items with low factor loading or cross-loading should have been deleted in the process of factor analysis, but the interviewees had a strong risk perception of these items, so these items were finally retained into the formal scale.

### 3.2. Exploratory Factor Analysis

By using SPSS 17.0 software, the EFA was conducted to further analyze the adequacy of scale items and to confirm the dimensional structure statistically.

#### 3.2.1. Data Gathering

The questionnaires were conducted on the principle of convenience sampling and judgment sampling. The 34 items in the pilot study were included in the scale of EFA and measured based on the five-point Likert scale. 1 means strongly disagree and 5 means strongly agree. That is to say, the bigger the value is, the greater the perceived risk is.

Some of the questionnaires were administered and returned on the spot, and others were filled out online. The independent sample *t*-test showed that two ways had no significant difference in demographic characteristics such as scale score, age, education background, income level, and blood donation experience. Finally, 981 questionnaires were returned, including 93 invalid questionnaires which were filled in optionally, missed key data, or were not filled out. So the overall response rate was 90.5%.

The sample information of EFA was as follows:

- Gender: male (45.2%), female (54.8%); age, 18 to 30 (39.1%), 31 to 40 (28.9%), 41 to 50 (17.0%), and 51 to 60 (15.0%).
- Educational background: junior high school level or lower (9.0%), high school level (30.7%), bachelor's degree (48.1%), and master's degree or above (11.9%).
- Income: below Ұ2000 (16.9%), Ұ2001 to Ұ4000 (38.4%), Ұ4001 to Ұ6000 (31.1%), and above Ұ6000 (13.6%).
- Profession: students (26.1%), soldiers (3%), teachers (5.7%), civil servants (10.5%), office workers (46.5%), and self-employers (8.0%).
- Blood donation experience: yes (15.9%), no (84.1%).

#### 3.2.2. Data Analysis and Results

The scale items were screened through heterogeneity test and correlation test. By calculating the correlation coefficient between single score and aggregate score, we found that the correlation coefficient between single scores of the second, twentieth, and thirty-fourth items and the aggregate score were less than 0.4, so these three items were included in deletion list. Meanwhile, the reliability analysis based on heterogeneity test showed that the coefficient of internal consistency (Cronbach's alpha) was 0.939, and if the twentieth and thirty-fourth items were deleted, Cronbach's alpha was above 0.939. Therefore, we finally deleted the second, twentieth, and thirty-fourth items.

Since the KMO value was 0.92 and the chi-square value of Bartlett's test was 4368.859 (*p* < 0.001), it indicated that the samples were suitable for EFA. There are multiple methods to conduct EFA, such as principal components analysis and principal axis factoring analysis. With the help of principal component analysis and the rotation method of maximum variance, 7 factors were extracted from 31 items and the 7 factors' eigenvalues were over 1, which could explain 61.956% of the total variance. By means of the principal axis factoring analysis and the rotation method of heterotrophic, 7 factors were also extracted. However, their structures were vague and could only explain 52.073% of the total variance. So the principal components analysis and the rotation method of maximum variance orthogonal were finally employed to extract factors in this study.

After the method of EFA was chosen, we deleted the items whose factor loading was less than 0.4 or among which apparent cross-loading existed so as to further simplify factor structures. The first EFA helped us delete the 1st, 4th, 6th, 7th, 10th, 12th, 18th, 22nd, 24th, 26th, and 33rd items. And in the process of the second EFA, the 8th, 9th, 11th, 13rd, 17th, 23rd, and 32nd items were deleted. The results of the third EFA showed that the factor loading of each item was above 0.5 and no apparent cross-loading existed. Meanwhile, we extracted three factors which could explain 61.271% of the total variances. Therefore, we concluded that the three factor structures were reasonable. According to the common characteristics of the items belonging to the three factors, the factors were named as trust risk, psychological risk, and health risk, respectively.

Trust risk could explain 21.209% of the total variance and was included in five items. Psychological risk could explain 20.286% of the total variance and was included in four items. And health risk could explain 21.209% of the total variance and was included in four items. The results showed that three-dimensional construct of perceived risk of NRBD was reasonable and the multidimensional construct developed by the grounded theory was also primarily verified statistically.  Table 4 summarized the results of EFA.

### 3.3. Confirmatory Factor Analysis

In order to develop a well-fitting measurement model, we conducted the CFA. CFA can test strongly the internal and external validity [27]. Firstly, we compared the single factor model, two-factor model (trust risk and psychological risk), and three-factor model (trust risk, psychological risk, and health risk), denoted, respectively, by M1, M2, and M3, to determine the best-fitting model. Then, we tested the reliability and validity of the best-fitting model.

#### 3.3.1. Data Gathering

The retained 13 items from the EFA were included in the scale of CFA and measured based on the five-point Likert scale. 1 means strongly disagree and 5 means strongly agree. That is to say, the bigger the value is, the greater the perceived risk is.

In the same way as the EFA, the data were gathered based on the principle of convenience sampling and judgment sampling. Some of questionnaires were administered and returned on the spot and others were filled out online. The independent sample *t*-test showed that the two ways had no significant difference in demographic characteristics such as scale score, age, education background, income level, and blood donation experience. 958 questionnaires were finally returned, including 106 invalid questionnaires which were filled in optionally, or missed key data, or were blank. So the overall response rate was 88.9%. In general, the number of samples must be ten times more than the number of freely estimated parameters in the structural equation model [28, 29], so the sample number of this study met the requirement.

The sample information of CFA was as follows:

- Gender: male (47.1%), female (52.9%); age, 18 to 30 (38.4%), 31 to 40 (29.1%), 41 to 50 (18.3%), and 51 to 60 (14.2%).
- Educational background: junior high school level or lower (8.6%), high school level (31.9%), bachelor's degree (55.3%), and master's degree or above (12.8%).
- Income: below Ұ2000 (16.9%), Ұ2001 to Ұ4000 (38.4%), Ұ4001 to Ұ6000 (31.1%), and above Ұ6000 (13.6%).
- Profession: students (23.0%), soldiers (3.3%), teachers (6.1%), civil servants (11.5%), office workers (47.1%), and self-employers (9.0%).
- Blood donation experience: yes (12.6%), no (87.4%).

#### 3.3.2. Data Analysis and Results

The CFA was conducted by using Lisrel 8.70 software. M1, M2, and M3 were compared to determine the optimal structural equation model, where M1 is a single factor model consisting of 13 observation variables, M2 is a two-factor model including trust risk and psychological risk, and M3 is a three-factor model including trust risk, psychological risk, and health risk. Considering that blood donation is nearly impossible to damage health and the perception of health risk may be caused by the public's distrust of blood donation, we merged health risk into trust risk in M2.

Table 5 summarized each fit index of the three models. Being consistent with the multidimensional construct developed by the grounded theory, comparison results showed that M3 was the most fitting one.

**Figure 3:**
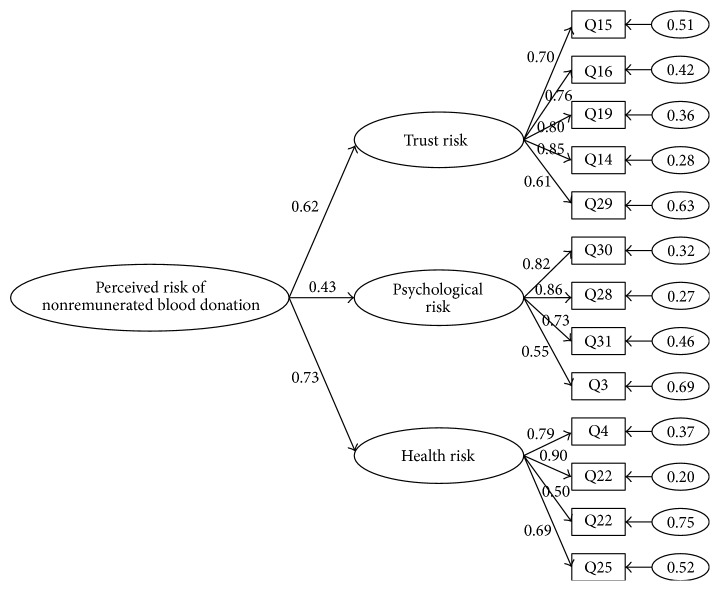
The results of CFA of Model 3.

As shown in Figure 3, the standardized factor loading of trust risk, psychological risk, and health risk corresponding to all the items was between 0.5 and 0.86, which demonstrated that the fit index of M3 was ideal. So we finally determined that the multidimensional construct of perceived risk of NRBD was a three-dimension one, including trust risk, psychological risk, and health risk.

Cronbach's alpha coefficient was used as the evaluation index of reliability. Cronbach's alpha coefficient of the whole scale was 0.84. And Cronbach's alpha coefficients of the three dimensions (i.e., trust risk, psychological risk, and health risk) were 0.833, 0.793, and 0.780, respectively. All Cronbach's alpha coefficients exceeded 0.7, the minimum limit of required performance, which proved that the internal consistency of the scale was within an acceptable range.

Composite reliability (CR) and average variance extracted (AVE) were used to evaluate the construct validity and convergent validity. Qiu and Lin [30] put forward that composite reliability, being similar to Cronbach's alpha, could calculate the proportion of variance of a measured variable. As shown in Table 6, the composite reliability of trust risk, psychological risk, and health risk was 0.86, 0.83, and 0.82, respectively, being greater than the recommended value 0.7 [31]. The findings showed that the three-dimensional construct of perceived risk of NRBD had good construct validity. Average variance extracted is an indicator of convergent degree of the latent variable being effectively estimated by a set of observed variables [31]. The average variances of the three dimensions were 0.56, 0.56, and 0.54, respectively, being greater than the recommended value 0.5 [32]. The results illustrated that each factor had a good convergent validity.

Finally, discriminant validity was examined. Qiu and Lin [30] suggested that the internal estimation of the correlation coefficient could be used to judge the discriminant validity. If 95% confidence intervals of two potential variable correlation coefficients do not contain 1, it indicates that the constructs have good discriminant validity. As shown in the third and fifth rows of Table 7, the 95% confidence intervals of each correlation coefficient do not contain 1, which meant that the correlation coefficients of the factors were not equal to 1 significantly. The results proved that the constructs had good discriminant validity.

Meanwhile, Fornell and Larcker [31] suggested that the discriminant validity could be tested by comparing the AVE with the square of correlation coefficient of two potential factors. As shown in the third row of Table 6 and the second and fourth rows of Table 7, the AVE were 0.56, 0.56, and 0.54, respectively, and the square of correlation coefficient was 0.068, 0.20, and 0.10, respectively, which all were less than the AVE. The findings further demonstrated that all the factors had good discriminant validity.

## 4. Conclusion and Discussion

In this paper, the multidimensional construct of NRBD perceived risk was preliminarily developed based on the grounded theory, the reasonableness of the multidimensional construct was verified through empirical analysis, and the measurement scale was finally determined. The qualitative and quantitative methods contributed to the results of three core dimensions, namely, trust risk, psychological risk, and health risk. We dissected the three dimensions of perceived risk of NRBD as follows and gave some implications for practice.

Firstly, trust risk reflects the absence of trust in blood donation. To be specific, it covers the public's questions about the need to pay for blood transfusion while blood is collected for free in NRBD; the worry about donated blood is used in illegal activities rather than in medical treatment and the dissatisfaction with the reimbursement procedure of donors' paid usage of blood transfusion service. Additionally, negative events such as Guo Meimei scandal also have an adverse effect on the social credibility of Chinese nonprofit organizations. Blood collection organizations in China are no exception. In the interview process, some participants mentioned that these negative events had a harmful impact on their intention of blood donation, which was also verified by our study. Besides, preferential policy cannot be completely enjoyed by blood donors. The costs of donors' blood transfusion cannot be reimbursed or the reimbursement procedure is very inconvenient. These phenomena undoubtedly damage the image and social credibility of blood collection organizations, thereby affecting the public's perceived trust. In order to reduce trust risk, more publicity must be given to NRBD. New media like Microblog or WeChat especially can contribute to the propaganda of the basic policies about NRBD and the reasonable charge rules so as to dispel public doubts. Once a negative event occurs, relevant organizations should take effective measures to clarify the misunderstanding via media and manage to regain the public's trust timely. As for preferential policies, relevant departments should simplify the reimbursement procedure. And a nationwide management information system for NRBD should be developed as soon as possible so as to make it easier for donors to enjoy preferential policy in any place.

Secondly, the dimension of psychological risk reflects the public's negative emotion about blood donation such as fear, tension, and anxiety. Obviously, blood donation is different from other types of donation, such as monetary donation. Because human body is the only source of blood and the Chinese public regards blood as the foundation of life, the public are wary of the negative effect of blood donation. Our study also showed that the public had a strong perception of pain and tension, and even the examination before the blood donation also made the public a bit of nervous. In addition, the opposition to blood donation from friends or families was a concern of potential donors. As for the corresponding managerial implications, blood collection organizations should implement timely and effective psychological intervention. For example, a comfortable donation environment or first-rate service is conducive to ease the public's tension and anxiety. On the other hand, the expertise and perfect service attitude of blood collection staff can physically and psychologically alleviate the public's pain to some extent. Meanwhile, blood collection organizations can run “circle marketing” campaign to promote the public's consciousness of NRBD and reduce social obstruction. In other words, the relevant institutions should take advantage of the social influence of blood donors who donate blood frequently or own high social status and implement special marketing programs to attract more blood donors.

Finally, health risk reflects the public's concerns about whether blood donation damages health or not. Our study found that the public pay close attention to sanitary conditions of blood collection facilities and the environment and believed that the facilities and environment were probably unsanitary and may catch infectious diseases. Meanwhile, the public also doubted the quality of blood collection facilities and feared that poor blood collection facilities would do harm to their health. In addition, the public deemed that physical examination before blood donation is too imprecise to determine whether donors could donate blood, which also harmed their health. Obviously, it is hardly possible for the public to perceive health risk. The high perceived health risk is caused by the lack of knowledge about blood donation. Given that, health education is undoubtedly an effective way to reduce perceived health risk. The authorities can spread the knowledge about blood donation through different channels, such as Microblog and WeChat, to help the public to dispel wrong understanding and to foster a right attitude toward blood donation.

Additionally, as shown in Table 7, the three dimensions of perceived risk of NRBD are closely related. So the corresponding reduction strategies of three dimensions could be adopted cooperatively so as to have a greatest effect on the alleviation of perceived risks.

## Figures and Tables

**Table 1:** An example of open coding.

Original data	Initial concepts
I think the nurses of blood station are unprofessional. I am afraid that they can't accurately find the location of the blood vessel.	A1-1 The nurses is unprofessional. A1-2 Blood donors get hurt.

And I doubt whether the facilities of blood collection are clean. The facilities may be used many times or used without sterilization, so donors might catch infectious diseases. It is difficult to timely detect some infectious diseases. It's too risky. I'd rather not donate blood.	A1-3 The equipment of blood collection is unsanitary. A1-4 The equipment of blood collection is reused. A1-5 The equipment of blood collection isn't sterilized.

The environment of blood donation is also a problem. For example, the air in blood donation vehicles isn't current and the seats of vehicles are dirty. In this environment, blood donors maybe catch infections from another.	A1-6 Catching some infections because of the equipment of blood collection. A1-7 The air of blood donation vehicle is not current. A1-8 The seats of blood donation vehicle is not cleaned frequently. A1-9 Blood is sold to make profit.

Lastly, I am worried that blood isn't used for medical treatment, but for making profit. It is said that blood stations staff benefit a lot through selling blood. The hospitals spend a lot buying blood from them, and these costs are paid by patients. Considering this possibility, I feel that I am cheated.	A1-10 Blood is sold to offer blood station staff high salary. A1-11 The fees for blood transfusion is too high. A1-12 Being cheated.

**Table 2:** Results of selective coding.

Core categories	Subcategories	Initial concepts
Trust risk	(1) Fear of blood waste in the process of usage	A2-1 When used in hospital, blood is wasted; A2-2 Too much blood transfusion for patients.
	(2) Fear of blood waste in the process of storage	A12-17 Too much blood is wasted because of improper storage; A11-7 Blood waste because of bad storage conditions.
	(3) Afraid of unprofessional nurses	A2-4 The nurses are not professional; A3-5 The nurses are lack of experience.
	(4) Fear of using blood to make profit	A1-10 Blood is sold to make profit; A1-11 Charge patients high price to make profit.
	(5) Afraid of excess of blood collection	A2-7 The staff of blood bank urges me to donate more blood; A3-8 Weigh blood inaccurately.
	(6) Fear of blood for other purposes	A5-9 Blood is used for experiment; A4-2 Blood is sold to blood products companies.
	(7) Fear of expensive blood for usage	A1-12 The fees of blood transfusion is too high, but blood collection is zero cost.
	(8) Fear of bad service attitude	A2-6 The staff is not hospitable; A3-7 The staff is too impatient to answer my questions.
	(9) Fear of complicated preferential blood usage procedure	A2-8 The usage procedure of blood is too complicated; A3-10 The usage procedure of blood in other places is more complex.
	(10) Suspicion about sterility of blood collection equipment	A5-16 Blood collection agencies use shoddy blood collection equipment to save cost; A7-2 The needle is reused.
	(11) Privacy disclosure	A3-6 Before blood donation private information is required to fill out, which can be disclosed; A4-5 It is boring to receive the messages from blood bank frequently.
	(12) The demand of blood is not really urgent	A9-6 Exaggerating the demand; A5-12 Appeals for blood donation is exaggerated.
	(13) Blood collection agencies cannot be trusted	A11-12 Blood collection agencies are not nonprofit; A6-13 Blood collection agencies cannot use productively blood.

Psychological risk	(14) Depressing clinical setting	A3-1 The atmosphere in the blood donation vehicle is very depressing.
	(15) Feeling pain	A3-11 I see needle and feel pain; A2-11 When I thought of drawing blood, I feel pain.
	(16) Feeling nervous	A3-9 I see red blood and feel nervous; A4-8 I am nervous because of bad mental quality.
	(17) Wasting time	A3-12 I have no time to donate blood; A4-7 It takes me too much time to donate blood.
	(18) Fearing physical examination before blood donation	A4-8 Screening test is unpleasant; A8-19 Physical examination is ominous.
	(19) Fear of opposition from her parents	A3-16 My parents think blood donation has negative impact on health; A9-22 My parent has attempted to stop me from donation.
	(20) Friends had bad experience	A10-11 My friend has told me donation is painful. A14-17 My friend has told me donation weakens health.
	(21) Afraid of seeing needle	A21-3 Dislike of needles; A17-8 The pain associated with needle.
	(22) Fear of discovering illness	A18-9 Fear of being revealing illness; A13-22 Discovering disease is heartbreaking.
	(23) Not having enough blood for donation	A19-11 My body is too small to donate; A17-10 Total blood volume is too small to donate.
	(24) Spend money in regaining health	A12-11 Spending money to supplement nutrients after donation.
	(25) Spend time in regaining health	A12-21 No time to regain health after donation; A18-13 Spending too much time to recover health.
	(26) Afraid of seeing blood	A17-16 A dislike of the sight of blood; A19-3 The sight of blood is ominous.
	(27) A dislike of trouble from inconvenient donation location	A18-7 I live far from the location of the blood donation center.
	(28) Feeling unbalance because of paid usage but unpaid donation	A12-8 Hard to understand blood usage is charged but unpaid donation; A9-7 It is unpleasant to be charged for blood usage.

Health risk	(29) Infection resulting from unsanitary equipment	A1-6 Catching infectious diseases because of the unsanitary blood collection equipment; A6-17 Contagion from unclean equipment.
	(30) Infection resulting from unsanitary environment	A1-19 Infection because of unclean the seat of blood donation vehicle; A9-12 Infection because of unsterilized donation site.
	(31) Infection resulting from shoddy equipment	A8-12 Using shoddy equipment result in infection; A1-23 Using low quality equipment lead to infection.
	(32) Infection resulting from improper disposal	A2-20 Infection resulting from improper disposal of pinhole after blood donation; A9-12 Lack of scientific guidelines after donation.
	(33) Simple examination before blood donation	A6-9 Simple examination cannot judge whether I am a qualified donor; A 9-10 Simple examination cannot guarantee my health.
	(34) Long-term consequence to health	A7-11 Having a negative impact on resistance to disease; A9-19 Body cannot recovery after donation.
	(35) Afraid of getting AIDS	A23-3 Blood donation may get AIDS; A13-16 Getting ADIS because of reused needle.
	(36) Wrong blood cross matching	A5-3 Wrong blood cross-matching cause life-threatening emergency.
	(37) Fear of being injured by the needle	A11-2 The pinhole is hard to heal up; A8-6 The needles is too thick, and must injury me.
	(38) Experiencing adverse symptoms after blood donation	A3-13 Feeling sleepy after blood donation; A4-13 Having a dull reaction after blood donation; A5-6 getting fat after blood donation.
	(39) Afraid of feeling dizzy in the process of blood donation	A11-1 I may feel dizzy in the process of blood donation; A8-7 Feeling dizzy because of my weak body.

**Table 3:** Literature comparison and verification.

Core categories	Subcategories	Literature comparison and verification
Trust risk	(1) Fear of blood waste in the process of usage	Chinese characteristic
	(2) Fear of blood waste in the process of storage	Chinese characteristic
	(3) Afraid of unprofessional nurses	[19]
	(4) Fear of using blood to make profit	Chinese characteristic
	(5) Afraid of excess of blood collection	[12]; Chinese characteristic
	(6) Fear of blood for other purposes	Chinese characteristic
	(7) Fear of expensive blood for usage	Chinese characteristic
	(8) Fear of bad service attitude	[19]
	(9) Fear of complicated preferential usage procedure of blood	Chinese characteristic
	(10) Suspicion about sterility of blood collection equipment	[13]
	(11) Privacy disclosure	Chinese characteristic
	(12) The demand of blood is not really urgent	[23]
	(13) Blood collection agencies cannot be trusted	Chinese characteristic

Psychological risk	(14) Depressing clinical setting	[19]
	(15) Feeling pain	[9]
	(16) Feeling nervous	[10]
	(17) Wasting time	[16]
	(18) Fearing physical examination before blood donation	[13, 18]
	(19) Fear of opposition from parents	Chinese characteristic
	(20) Friends had bad experience	[24]
	(21) Afraid of seeing needle	[10]
	(22) Fear of discovering illness	[13]
	(23) Not having enough blood for donation	[12]; Chinese characteristic
	(24) Spend money in regaining health	Chinese characteristic
	(25) Spend time in regaining health	Chinese characteristic
	(26) Afraid of seeing blood	[17, 19]
	(27) A dislike of the trouble from inconvenient location	[12]; Chinese characteristic
	(28) Feeling unbalance because of paid usage but unpaid donation	Chinese characteristic

Health risk	(29) Infection resulting from unsanitary equipment	[13]
	(30) Infection resulting from unsanitary environment	Chinese characteristic
	(31) Infection resulting from shoddy equipment	Chinese characteristic
	(32) Infection resulting from improper disposal	[19]
	(33) Simple examination before blood donation	Chinese characteristic
	(34) Long-term consequence to health	[12]; Chinese characteristic
	(35) Afraid of getting AIDS	[14]
	(36) Wrong blood cross matching	Chinese characteristic
	(37) Fear of being injured by the needle	[6, 9]
	(38) Experiencing adverse symptoms after blood donation	[12]; Chinese characteristic
	(39) Afraid of feeling dizzy in the process of blood donation	[9]

**Table 4:** Results of EFA.

Items	Factors		
	Trust risk	Psychological risk	Health risk
Q15 Free blood must be paid by patients, which is unfair.	0.727		
Q16 The usage of blood is uncovered. There is no idea about whether the blood is used in clinical treatment.	0.717		
Q21 The blood isn't used for clinical treat, but is sold, transferred or is used in other illegal activities.	0.701		
Q19 The blood is used for making illegal profit by blood collection and supply institutions.	0.699		
Q14 Complicated procedure makes it difficult for donors to enjoy preferential policy.	0.677		
Q29 I will feel painful when I think of blood donation.		0.849	
Q30 I will be nervous when I think of blood donation.		0.790	
Q28 I am afraid of the examination before blood donation.		0.746	
Q31 My friends and families are against my blood donation.		0.686	
Q3 Unsanitary blood collection facilities may cause infection.			0.846
Q4 Unsanitary blood collection conditions may cause infection.			0.835
Q22 Simple examination before blood donation cannot guarantee whether donors are qualified.			0.651
Q25 Poor blood collection facilities may harm donors' health.			0.581
Eigenvalues	2.757	2.663	2.545
Percentage of variance explained (%)	21.209	20.486	19.579
Cumulative percentage of variance explained (%)	21.209	41.695	61.274

**Table 5:** Fit index.

Model	Chi-square	df	Chi-square/df	RMSEA	GFI	NFI	IFI	CFI	NNFI
M1	3087.16	260	11.87	0.23	0.64	0.69	0.71	0.71	0.65
M2	1804.28	256	7.05	0.16	0.78	0.82	0.84	0.84	0.81
M3	1117.04	248	4.50	0.08	0.84	0.89	0.91	0.91	0.89

All chi-square tests are statistically significant. RMSEA, root mean square error of approximation; GFI, goodness of fit tests; NFI, normed fit index; IFI, incremental fit index; CFI, comparative fit index; NNFI, nonnormed fit index.

**Table 6:** CR and AVE of Model 3.

Dimension	Trust risk	Psychological risk	Health risk
CR	0.86	0.83	0.82
AVE	0.56	0.56	0.54

CR, composite reliability; AVE, average variance extracted.

**Table 7:** Discriminant validity.

	Trust risk	Psychological risk
Psychological risk		
*r* (*r* ^2^)	0.26^*∗∗∗*^ (0.068)	
95% CI	(0.19, 0.33)	
Health risk		
*r* (*r* ^2^)	0.45^*∗∗*^ (0.20)	0.31^*∗∗*^ (0.10)
95% CI	(0.39, 0.51)	(0.24, 0.38)

^*∗∗*^ *p* < 0.01;  ^*∗∗∗*^ *p* < 0.001. CI, confidence interval.
